# The role of lipid droplet associated proteins in inherited human disorders

**DOI:** 10.1002/1873-3468.14779

**Published:** 2023-12-14

**Authors:** Xiaowen Duan, David B. Savage

**Affiliations:** 1 University of Cambridge Metabolic Research Laboratories, Wellcome Trust-MRC Institute of Metabolic Science, Cambridge, CB2 0QQ, UK

## Abstract

**Figure:**
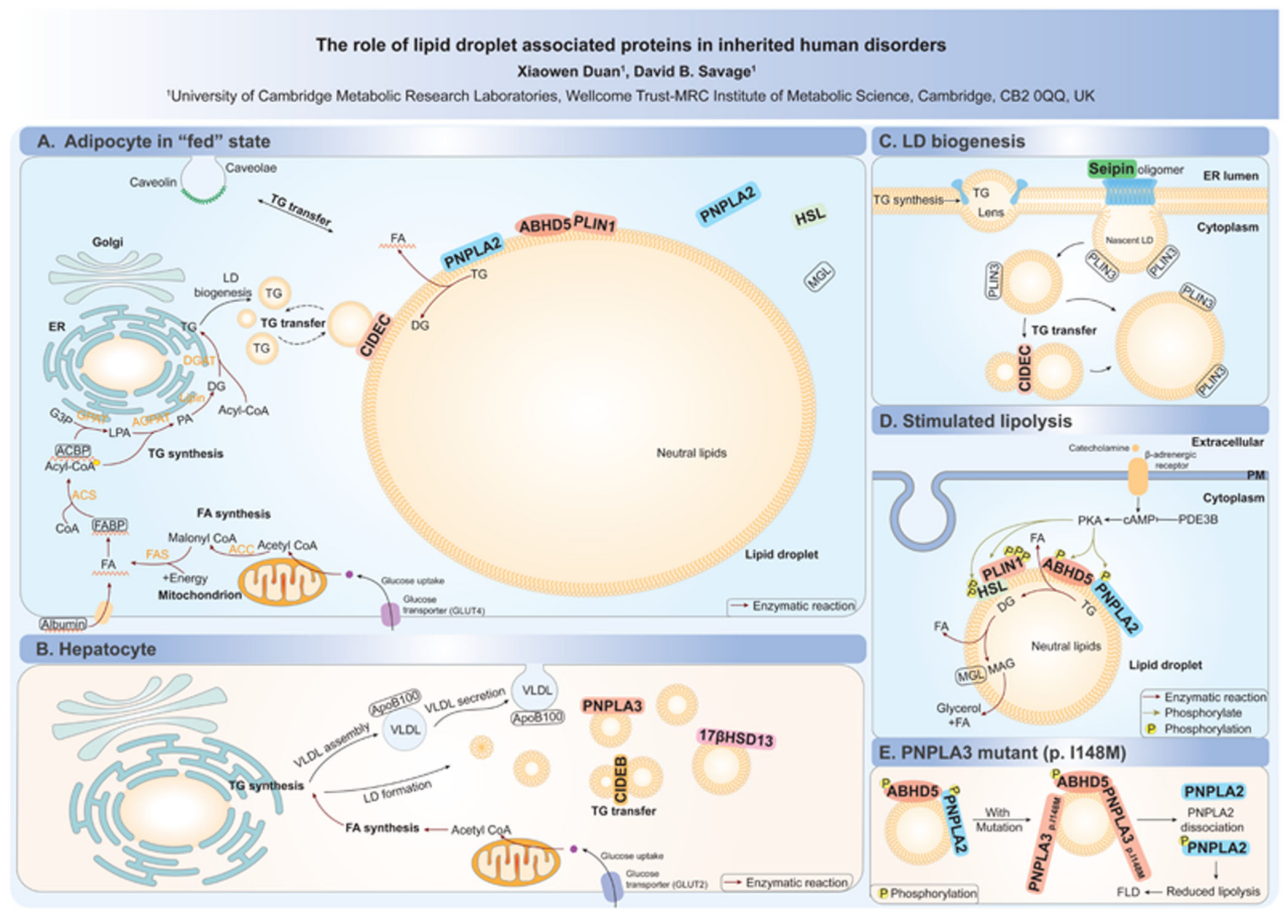

Lipid droplets (LDs) are evolutionarily conserved organelles which can form in almost any cell type. They enable the compartmentalization of hydrophobic neutral lipids within the aqueous cytosol. Despite the variation in number, size and composition of LDs in different cells, all LDs share a uniform structure with an amphipathic phospholipid (PL) monolayer enclosing a neutral lipid core. LDs emerge from the ER with the composition of the PL monolayer reminiscent of the ER bilayer [1]. A variety of proteins, intimately involved in regulating lipid flux into and out of the droplet, associate with the LD surface PL monolayer through hydrophobic hairpins [2, 3], amphipathic helices [4–7] and fatty acid modifications [8–10]. For example, LD growth and degradation are tightly controlled by proteins/enzymes localised on the surface monolayer regulating triacylglycerol (TG) synthesis and hydrolysis. In addition to their roles in surplus energy storage, membrane synthesis and lipid signaling, LDs play a crucial role in relieving cellular stresses including lipotoxic stress [11–13], ER stress [14] and oxidative stress [15, 16].

LD biology is linked to key physiological processes and their dysregulation is associated with several human diseases [1, 17, 18]. In some instances, changes in the number and/or size of LDs in the context of disease are almost certainly secondary to the disease initiating event. For example, the number and size of LDs in hepatocytes will increase in circumstances where excess energy in the form of fatty acids and/or carbohydrate (glucose or fructose) is delivered to the liver as occurs in obesity. Similarly a defect in lipoprotein secretion [19] as might occur in patients with mutations affecting apolipoprotein B (abetalipoproteinaemia) [20, 21] or in mitochondrial fatty acid oxidation [22–24] will lead to liver steatosis. Many other cell types similarly manifest changes in LDs in the context of various diseases.

In contrast to this type of ‘secondary’ change in LD morphology, in this short graphical review we highlight human diseases linked to genes encoding proteins known to be directly physically associated with LDs whose primary action relates at least in part directly to the LD. These are briefly summarized in Table 1.

Perhaps the most striking example of a disease state directly linked to LD perturbation is **lipodystrophy** [25]. Lipodystrophies are characterized by a generalized or partial (regional) lack of body fat^1^. In partial lipodystrophies where adipocytes are retained, they also appear to be dysfunctional in many but not all instances. The paucity of functioning adipose tissue is almost always associated with secondary ectopic fat accumulation [27], a problem exacerbated by hyperphagia which is present in many patients with lipodystrophy due to the associated relative or near total leptin deficiency[27, 28]. This in turn leads to insulin resistance and almost all the other metabolic co-morbidities typically associated with obesity [28]. More than 20 genes have now been linked to human lipodystrophies [25, 29, 30]. These include key transcriptional regulators of adipogenesis such as *PPARG*, nuclear envelope proteins thought to impact gene expression (*LMNA*), proteins implicated in lipid trafficking into adipocytes (*CAV1, PTRF*) and a protein directly involved in TG synthesis (*AGPAT2*) – in these settings, the paucity of fat is associated with corresponding changes in LDs, but these largely appear to be secondary to the primary perturbation so are not discussed further herein. However, a subset of lipodystrophies do primarily involve proteins directly associated with LDs. These include *BSCL2, PLIN1, LIPE* and *CIDEC* - their normal biological roles and associations with specific lipodystrophy subtypes are summarized in Table 1.

**Neutral lipid storage disease (NLSD)** is characterized by LD accumulation in several tissues including skin, heart, skeletal muscle, liver, central nervous system, and leukocytes [31, 32]. It is caused by autosomal recessive loss-of-function mutations in *PNPLA2* (*ATGL*) which encodes the rate-limiting enzyme in lipolysis or by loss-of-function mutations affecting *ABHD5*, a key activator of *PNPLA2* [33]. An important clinical difference in the disease manifestations of these disorders is the presence of ichthyosis (scaly, itchy red skin) in cases caused by *ABHD5* mutations [34]. This is thought to relate to disruption of the role of *ABHD5* in activating PNPLA1 to catalyse the esterification of ω-hydroxyceramides with linoleic acid in skin cells [35].

**Non-alcoholic fatty liver disease (NAFLD)** is characterized by abnormal accumulation of TG-containing LDs in the liver and affects almost one third of the population [36]. It most commonly occurs in the setting of suboptimal lipid storage in adipose tissue, but can also follow impaired VLDL secretion from the liver [37], impaired fatty acid oxidation [38] and HCV infection which appears to stabilize LDs [39]. However, there are a couple of instances in which variants in genes encoding LD proteins have been linked to NAFLD, a good example being the *PNPLA3* gene [40]. In this instance, one specific missense variant (p.I148M) is associated with an increased risk of NAFLD. Functional studies suggest that this variant enhances PNPLA3 accumulation on the surface of LDs, where it competitively inhibits the activation of PNPLA2 by ABHD5, thereby inhibiting PNPLA2 dependent lipolysis [41, 42]. A loss-of-function splice variant in another LD associated protein HSD17B13 [43] was reported to protect against progression from steatosis to steatohepatitis [44, 45]. More recently, a multistage exome sequencing and genetic association analysis showed that variants in *CIDEB*, which encodes a structural protein found in hepatic LDs, had a protective effect against fatty liver disease [46]. CIDEB is predominantly expressed in hepatocytes where it appears to perform a similar function to CIDEC.

## Supplementary Material

Graphical review

## Figures and Tables

**Table 1:** 

Gene/protein name and disease inheritance pattern	Disease	Putative function
*BSCL2* (Bernardinelli-Seip congenital lipodystrophy type 2) /Seipin; Autosomal recessive	CGL2, Congenital generalized lipodystrophy type 2 [47]. A severe generalized form of lipodystrophy associated with severe insulin resistance, early onset diabetes, hypertriglyceridaemia and NAFLD.	Integral ER membrane protein which homo-oligomerises at sites from which LDs emerge from the ER [48, 49]; Thought to be intimately involved in LD biogenesis [50, 51]; Has also been shown to be required for adipogenesis though the mechanism for this action remains unclear [52].
*PLIN1*/Perilipin-1; Autosomal dominant	FPLD4, Familial partial lipodystrophy type 4 [53]. Characterized by childhood or young adult onset of loss of subcutaneous adipose tissue primarily affecting the lower limbs, with diabetes, hypertriglyceridemia, hypertension and NAFLD [53, 54]. However, gene-burden testing has also suggested that some loss-of-function frameshift mutations are associated with a favorable waist-hip ratio [55–57].	Selectively expressed in adipocytes where it constitutively localizes on LDs by means of a series of amphipathic helices [6, 58]; Key regulator of basal and stimulated lipolysis; Phosphorylated by PKA [59] and then associates with activated HSL; Indirectly regulates PNPLA2 activity by sequestering ABHD5 in the ’fed’ state [60].
*LIPE/* Hormone sensitive lipase (HSL); Autosomal recessive	FPLD6, Familial partial lipodystrophy type 6 [61, 62]. Abnormal subcutaneous fat distribution with variable excess fat accumulation on upper body and reduction of fat from the lower extremities with progressive adult-onset myopathy and variable association with diabetes, hypertriglyceridaemia and NAFLD [63, 64].	Recruited to LDs following PKA-mediated phosphorylation of HSL and perilipin in response to β-adrenergic stimulation [65]; Primarily functions as a DG lipase [66, 67].
*CIDEC* (Cell death-inducing DFFA-like effector C)/ Lipid transferase CIDEC; Autosomal recessive (only 1 patient with this condition described to date)	FPLD5, Familial partial lipodystrophy type 5 [68]. Partial lipodystrophy manifesting as muscular lower limbs and acanthosis nigricans with diabetes, severe hypertriglyceridemia and secondary pancreatitis.	Enriched in white adipocytes where it is located at contact sites between LDs [69]; Facilitates directional neutral lipid transfer from the smaller to the larger droplet [69]; Required for the formation of large unilocular LDs in white adipocytes [70] [71, 72]
*PNPLA3* (Patatin-like phospholipase domain-containing protein 3)/ 1-acylglycerol-3-phosphate O-acyltransferase PNPLA3	FLD1, Fatty liver disease [40, 73–76]. A prevalent *PNPLA3* missense variant (p. I148M) is associated with an increased risk of hepatic steatosis.	Shows TG hydrolase activity in vitro [77] and is usually present at low levels on the surface of LDs where it competes with PNPLA2 for association with ABHD5 [78], thereby inhibiting lipolysis [41]; The I148M variant increases PNPLA3 stabilisation on LDs and thus inhibits lipolysis [41, 42].
*HSD17B13*/17-β- HSD13	FLDP, Fatty liver disease, protection from [44, 79, 80]. A splice site variant is associated with protection against the progression to chronic liver disease from simple steatosis.	Enriched in hepatocytes and localises on LDs [80]; Enzymatic substrates remain unclear [44]; Overexpression increases SREBP1 expression and TG content in mouse liver [45];
*CIDEB* (Cell death-inducing DFFA-like effector B)/ Lipid transferase CIDEB	Gene-burden testing suggests that loss-of-function variants reduce liver steatosis and protect against liver disease of any cause [46].	Similar to CIDEC but enriched in hepatocytes [46, 81]; *Cideb*-null mice were protected against fatty liver [82].
*PNPLA2* (Patatin-like phospholipase domain-containing protein 2)/ Patatin-like phospholipase domain-containing protein 2 (also known as Adipose triglyceride lipase (ATGL); Autosomal recessive	NLSDM, Neutral lipid storage disease with myopathy [83–85]. Characterized by the accumulation of of TG-containing LDs in cells/tissues including leukocytes, skin and muscle. This is then associated with adult onset progressive proximal muscle weakness, and about 50% of patients develop cardiomyopathy. Variable association with diabetes, hepatic steatosis and hypertriglyceridemia [84].	A triglyceride lipase that localizes to LDs in response to beta-adrenergic activation and association with ABHD5 [86]; Catalyzes the initial step in TG hydrolysis [87].
*ABHD5/* 1-acylglycerol-3-phosphate O-acyltransferase ABHD5; Autosomal recessive	Chanarin-Dorfman syndrome/NLSDI, Neutral lipid storage disease with ichthyosis [34]. Characterized by intracellular TG-containing LDs present in many tissues. In contrast to NLSD, patients also manifest a non-bullous erythrodermic form of ichthyosis [34].	Localizes at the surface of LDs [88], acting as a coactivator of PNPLA2 to promote TG hydrolysis [86]: Also acts as an acyltransferase for phosphatidic acid synthesis [89].

## Footnotes

1 Localised forms also exist but these are not considered further here as they are not directly linked to proteins involved in lipid droplet metabolism ^26. Hussain, I. and A. Garg, *Lipodystrophy* *Syndromes*. Endocrinol Metab Clin North Am, 2016. **45**(4): p. 783-797.^.

## Abbreviations

Text:

LD: Lipid droplet
ER: Endoplasmic Reticulum
TG: Triacylglycerol
NLSD: Neutral lipid storage disease
NAFLD: Non-alcoholic fatty liver disease
DG: Diacylglycerol

Figure:

FA: Fatty acid
GLUT4: Glucose transporter type 4
ACC: Acetyl-CoA carboxylase
FAS: Fatty acid synthase
FABP: Fatty acid-binding protein
ACS: Fatty acyl-CoA synthetase
ACBP: Acyl-CoA-binding protein
G3P: Glycerol-3-phosphate
GPAT: Glycerol-3-phosphate acyltransferase
LPA: Lysophosphatidic acid
AGPAT: 1-acylglycerol-3-phosphate-O-acyltransferase
PA: Phosphatidic acid
Lipin: Phosphatidate phosphatase
DGAT: Diacylglycerol O-acyltransferase
GLUT2: Glucose transporter type 2
VLDL: Very low density lipoprotein
PM: Plasma membrane
PKA: Protein kinase A
MAG: Monoacylglycerol
