# Supplementary material for: The role of lipid droplet associated proteins in inherited human disorders
Source: FEBS Lett. Author manuscript; Available in PMC 2025 Jan 25. (PMC7617339; doi:10.1002/1873-3468.14779)

The role of lipid droplet associated proteins in inherited human disorders

Xiaowen Duan[1], David B. Savage[1]

[1]University of Cambridge Metabolic Research Laboratories, Wellcome Trust-MRC Institute of Metabolic Science, Cambridge, CB2 0QQ, UK

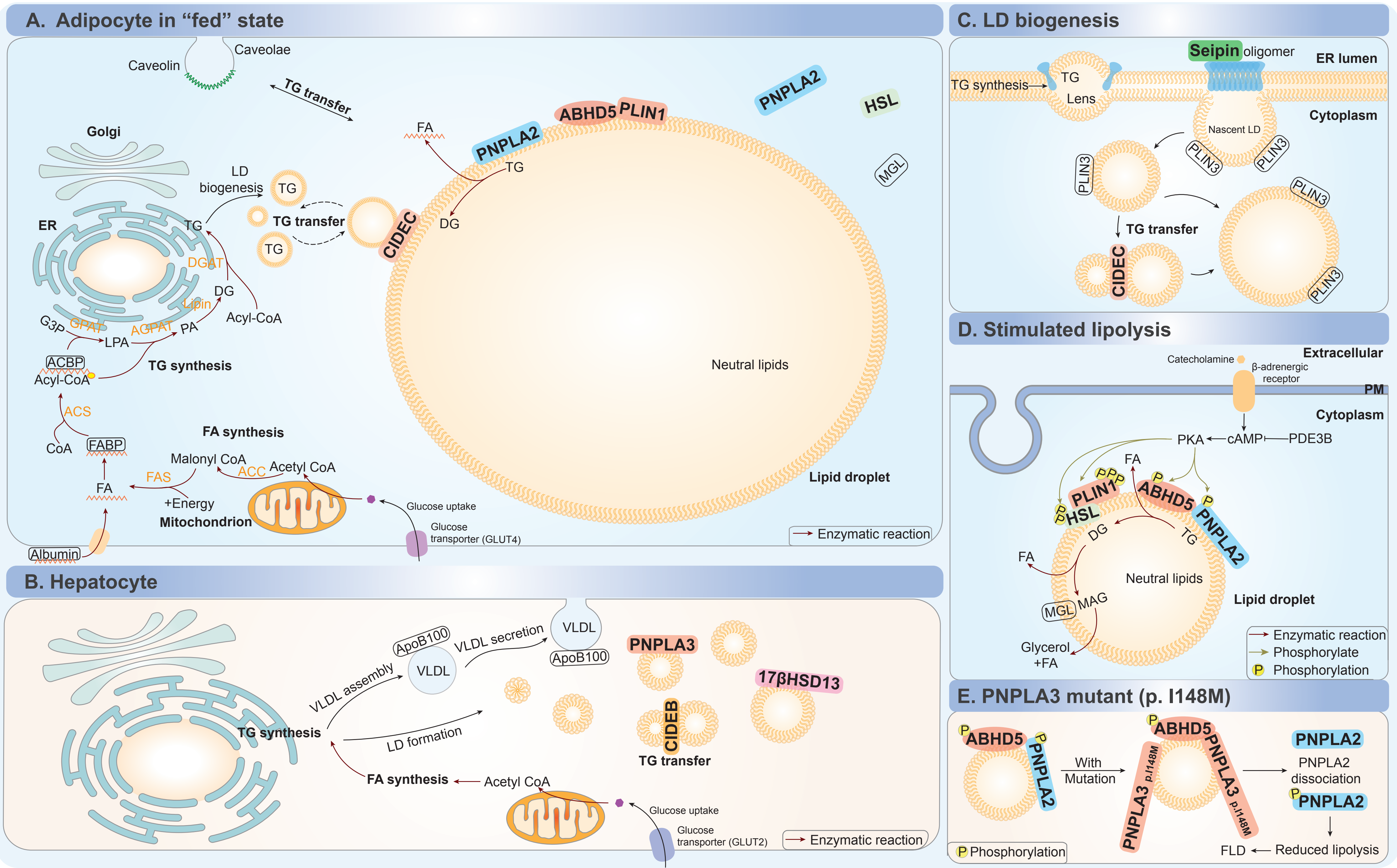

Supplement: Graphical review [file EMS202141-supplement-Graphical_review.pdf]
